# The role of prezygotic isolation mechanisms in the divergence of two parasite species

**DOI:** 10.1186/s12862-016-0799-5

**Published:** 2016-11-09

**Authors:** Tina Henrich, Martin Kalbe

**Affiliations:** Department of Evolutionary Ecology, Max Planck Institute for Evolutionary, Biology, August-Thienemann-Strasse 2, 24306 Plön, Germany

**Keywords:** Reproductive isolation, Hybridization, Mate choice, *Schistocephalus solidus*, *Schistocephalus pungitii*, Sticklebacks

## Abstract

**Background:**

The formation of reproductive barriers in diverging lineages is a prerequisite to complete speciation according to the biological species concept. In parasites with complex life cycles, speciation may be driven by adaptation to different intermediate hosts, yet diverging lineages can still share the same definitive host where reproduction takes place. In these cases, prezygotic isolation mechanisms should evolve very early and be particularly strong, preventing costly unfavourable matings.

In this study, we investigated the importance of prezygotic barriers to reproduction in two cestode species that diverged 20–25mya and show an extraordinary degree of specificity to different intermediate hosts. Both species share the same definitive hosts and hybridize in the laboratory. Yet, natural hybrids have so far not been detected.

**Methods:**

We used a combination of different experiments to investigate the role of prezygotic barriers to reproduction in the speciation of these parasites. First, we investigated whether hybridization is possible under natural conditions by exposing lab-reared herring gulls (*Larus argentatus*, the definitive hosts) to both parasites of either sympatric or allopatric combinations. In a second experiment, we tested whether the parasites prefer conspecifics over parasites from a different species in dichotomous mate choice trials.

**Results:**

Our results show that the two species hybridize under natural conditions with parasites originating either from sympatric or allopatric populations producing hybrid offspring. Surprisingly, the mate choice experiment indicated that both parasite species prefer mates of the different species to conspecifics.

**Conclusions:**

Neither fundamental constraints against hybridization in a natural host nor assortative mate choice sufficiently explain the persistent segregation of the two tapeworm species in nature. Hence, postzygotic ecological selection against hybrids is presumably the more important driving force limiting gene flow between the two parasite sister species.

**Electronic supplementary material:**

The online version of this article (doi:10.1186/s12862-016-0799-5) contains supplementary material, which is available to authorized users.

## Background

The initiation of barriers to gene flow is a crucial step in the course of speciation [1]. Following the biological species concept [2], diverging lineages can only complete speciation if gene flow is eventually restricted or eliminated. The formation of barriers can be either adaptive or a by-product that arises through genetic drift or the epistatic interactions of genes that increase the amount of genetic differences between lineages. Species boundaries arising this way do not only allow adaptation of each lineage to specific conditions but also prevent wasting resources in unfavourable matings.

Barriers to reproduction can be effective before and after the formation of a zygote. Prezygotic barriers include spatial or temporal separation of reproduction as well as behavioural differences leading to sexual isolation. Prezygotic barriers are arguably the most important and effective barriers, given that they would act early in the life cycle of an organism, impose the strongest impediment to gene flow, and could prevent costly but unfavourable mating combinations [3–6]. Selection against hybrids may lead to reinforcement, thereby increasing reproductive isolation of the parental species [7].

Components of postzygotic reproductive isolation include intrinsic hybrid inviability (e.g., Dobzhansky-Muller incompatibilities) as well as extrinsic lower fitness of hybrids (i.e., natural or sexual selection against hybrids). Speciation is a non-linear process and hybrid inviability can evolve faster in some taxa than in others due to differences in regulatory evolution leading to a higher probability of incompatibilities [8]. Different genetic architecture and alternative developmental pathways [9, 10] also contribute to hybrid inviability so that not only divergence time and correlated sum of genetic differences between two lineages contribute to intrinsic postzygotic isolation [8].

It is also likely that reproductive isolation is often caused by the interaction and accumulation of multiple pre- and postzygotic barriers [11–14]. The strength of each barrier is variable among taxa and may also change through the speciation process [15]. The order in which reproductive barriers evolve is likely variable among taxa [15], making it difficult to predict the evolution of reproductive isolation in general. However, Coyne and Orr [16, 17] demonstrated in a large dataset on *Drosophila* species pairs that the strength of divergence is related to the strength of pre- and postzygotic isolation. Furthermore, prezygotic isolation evolves faster than postzygotic isolation and prezygotic isolation is higher in sympatric than allopatric species pairs.

Speciation in parasites has not received the same attention as speciation in non-parasitic species, despite the fact that parasites are more abundant than non-parasites [18, 19]. However, some specific features of parasitic species make them an interesting study system for speciation. For example, the hosts represent a special case of a living environment which imposes a great diversity of selection factors, leading to very elaborate adaptations, large variety of reproductive tactics (including hermaphrodism, asexual reproduction and mixed mating systems), often short generation times and largely fragmented populations [20]. For parasites, divergent selection can act through the specialization to specific host species. The host can be regarded as a very dynamic environment that requires constant adaptation of the parasite (i.e., for food exploitation or evasion of the immune system). In many cases, adaptations to different host species will also be associated with a spatial isolation that prevents reproduction between different lineages.

In general, there are two possibilities for parasite speciation through host specificity [21–23]: through host-switching or through congruent co-speciation. Host-switching requires an initial decrease in host specificity for the parasite in order to be able to establish in the new host, followed by a compulsory subsequent increase in host specificity, which distinguishes host switching from host range expansion. Congruent co-speciation follows the speciation of the host lineages, where parasites and hosts exhibit congruent phylogenies, a process which is described as Fahrenholz’s rule [24]. Co-speciation has primarily been shown between lice and their hosts [25–28]. These cases involve host specificity on the single host on which also mating occurs, therefore creating the possibility for allopatric speciation.

In parasites with complex life cycles however, sexual reproduction with the possibility of cross-species mating does not necessarily occur during all stages of the life cycle making parasite speciation through host specificity more convoluted. Host specificity can differ between the stages of the life cycle and may depend on the function of the host at a specific stage, which also includes the definitive host where sexual reproduction occurs. If hosts mainly serve as transportation vehicles, a lower specificity may be advantageous, whereas hosts that are severely exploited for nutrients at other parasite life stages require a closer adaptation, favoring higher specialization by the parasite [29, 30]. The possibility for host switching events or co-speciation to initiate speciation in parasites therefore strongly depends on the position of the host in the parasite’s life cycle. Host switching events on any level of intermediate hosts might not lead to speciation at all, if reproductive isolation is not selected for in the definitive host.

The role of prezygotic isolation mechanisms in speciation through host switching or co-speciation on the level of intermediate hosts is not clear, as this does not automatically infer spatial or temporal isolation of reproduction in the definitive hosts. In this case, postzygotic barriers to reproduction may be a more important factor driving speciation.

To investigate the importance of prezygotic isolation mechanisms in a parasite with complex life cycle, we used a species pair of cestodes: *Schistocephalus solidus*, specific to three-spined sticklebacks (*Gasterosteus aculeatus*) and *Schistocephalus pungitii*, specific to nine-spined sticklebacks (*Pungitius pungitius*). Both are highly host-specific on the level of the second intermediate host (the two stickleback species) with each species only being able to infect this single fish species [31, 32]. Host specificity may have played an important role as the major selective force in this divergent speciation, as the interaction with the stickleback intermediate host – particularly its immune system - is highly specific and crucial for the parasite’s fitness [33, 34].

Nishimura et al. [35] suggested that speciation occurred shortly after the divergence of the two stickleback species as a single event, which would argue for a co-speciation following the divergence of the stickleback species. The strong separation indicated by molecular data suggests low or no gene flow between these two parasite species, even though extensive studies on this have not yet been conducted. However, we were recently able to show that the two parasite species can still produce viable hybrids in the laboratory [36] which show an increased host range being able to infect both fish hosts. This study did not identify any obvious fitness disadvantages for several stages of the parasite life cycle (hatching rate, infection rate in first and second intermediate hosts as well as performance in the second intermediate host). Nonetheless, the reproductive fitness of F1 hybrids was not assessed in that study and the possibility that fitness disadvantages could become apparent only after the first generation of hybrids remains.

Even though the two cestode species successfully interbreed under laboratory conditions, so far there is no evidence for hybridization from natural populations [35]. This is particularly surprising because an expansion of the extremely narrow second intermediate host range is presumably highly advantageous for the parasite’s chance of developing into a plerocercoid infective to the definitive bird host. Hence, it appears quite likely that selection against hybridization of the two parasite sister species takes place earlier in their complex life cycle.

We therefore studied whether prezygotic barriers are responsible for the anticipated low amount of gene flow in natural populations. For this purpose we investigated whether i) there are incompatibilities for hybridization in sympatric populations ii) there is spatial isolation within the definitive hosts preventing hybridization or iii) if there is preferential mating with conspecifics in a mate choice experiment.

## Methods

### Study organisms

*Schistocephalus solidus* and *S. pungitii* are closely related cestodes with a complex life cycle involving three different hosts [37, 38]. Both species use piscivorous birds as definitive hosts, where the adult worms reproduce sexually. These parasites are simultaneous hermaphrodites, mature rapidly and usually complete the reproduction within one week in the definitive host [38, 39]. The eggs are then released with the bird’s feces into the water, where they hatch into free-swimming larvae. These larvae have to be eaten by cyclopoid copepods, the first intermediate hosts, to develop into procercoids. If infected copepods are eaten by sticklebacks, the second intermediate hosts, the parasite migrates through the gut wall into the fish’s body cavity and develops into a plerocercoid. Both parasites are highly specific on this level of the life cycle: *S. solidus* can only infect three-spined sticklebacks (*Gasterosteus aculeatus*), while *S. pungitii* is only infective to nine-spined sticklebacks (*Pungitius pungitius*). Several experiments have already investigated this phenomenon of high host specificity [31, 32] while hybrids of the two parasites species show an expanded host range and are able to infect both stickleback species [36]. The life cycle is completed when piscivorous birds prey upon infected sticklebacks.

The life cycle for both cestodes can be maintained in the lab by replacing the definitive host with an artificial breeding system [40, 41]. For this purpose, worms are placed into sealed net bags and incubated in a 40 °C warm culture medium (for details see [41]) in the dark for a time period of up to 8 days, where most of the egg production is accomplished [38, 39]. The eggs are then washed and stored in tap water at 4 °C before development is induced. Hatching can be triggered and mostly synchronized by exposure to light, which facilitates experimental exposure of both copepods and later on, fish.

### Natural hybridization in allopatric and sympatric species pairs

To test whether spatial constraints inhibit either parasite from hybridization in natural definitive hosts and if hybridization of *S. solidus* and *S. pungitii* from a sympatric population was possible in principle, we infected two herring gulls (*Larus argentatus*) with eight plerocercoids each (four *S. solidus* and four *S. pungitii*). One gull was exposed to eight cestodes of the two species that both originated from a sympatric population in Obbola, Sweden (63° 39′ N, 20° 17′ E). The second gull was exposed to four lab-bred cestodes of *S. solidus* from a population in Skogseidvatnet, Norway (60° 14′ N, 5° 55′ E) and four cestodes of *S. pungitii* from wild caught nine-spined sticklebacks from Lebrader Teiche, Germany (54° 22^′^ N, 10° 42′ E). There is no population of nine-spined sticklebacks that could harbor *S. pungitii* in the vicinity to the population of three-spined sticklebacks from Skogseidvatnet, Norway. Consequently this *S. solidus* population is very unlikely to encounter *S. pungitii* and hence there is no selection for a barrier to hybridization, whereas mating barriers are more likely to arise in places where both parasite species occur in sympatry and the possibility of reinforcement is given.

Feces of the herring gulls were collected between 24 and 72 h after infection. Eggs were washed and incubated at 20 °C in the dark for three weeks before coracidia were hatched [38]. 96 hatched coracidia were collected from the feces of each gull and typed using five different microsatellite markers [42], which are used for species discrimination as well as the identification of hybrids [36].

### Mate choice experiment

In a dichotomous-choice experiment, we determined the mating preference of a focal cestode that could choose between a conspecific and a cestode from a different species over 48 h. Both *S. pungitii* and *S. solidus* cestodes were used as focal worms in choice and control trials. *S. pungitii* plerocercoids originated from field-collected *P. pungitius* caught at Lebrader Teiche, Germany. *S. solidus* plerocercoids were obtained from lab-infected *G. aculeatus* (12 weeks post exposure) and those originated either from a population from lake Skogseidvatnet, Norway or Xinzo de Limia, Spain (42° 07′ N, 07° 39′ W). We chose *S. solidus* cestodes from two different populations to ensure that those plerocercoids were derived from different families. As it has been shown earlier that *S. solidus* prefers closely related over distantly related mates [43], we wanted to ensure that this does not affect our experiment. The prevalence of *S. pungitii* in this large *P. pungitius* population is rather high (est. 10–20 %). We therefore assumed that the probability of accidentally selecting two closely related cestodes with a random sample from the field is negligible.

**Fig. 1:**
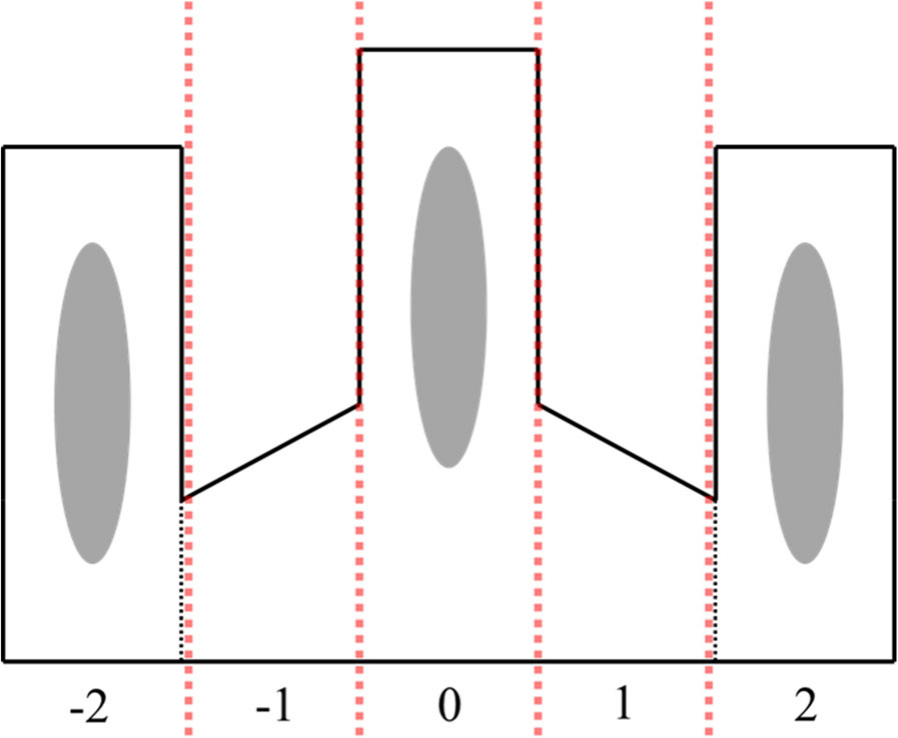
Setup for mate choice experiment (modified after Lüscher and Wedekind, 2002). A focal worm was placed together with two stimulus worms into a fork-shaped mesh bag in a culture medium mimicking the situation in the bird’s gut. The focal worm (middle compartment) can move freely between all compartments, while the stimulus worms are restricted to side compartments that overlap the middle compartment. Scores were assigned according to the focal worm’s position. “0” was considered neutral, 1 & 2 (or −1 & -2 respectively) for a tendency to the side of one of the stimulus worms. If there was a minimum of 25 % overlap between the focal worm and one of the stimulus worms, we assigned the score “3” or “-3”, as this was a position where mating was possible

The sticklebacks were killed with an overdose of MS222 (tricaine methanesulfonate, 1 mg/ml) followed by a cervical incision. Afterwards the plerocercoids were removed from the fish, and weighed to the nearest 0.01 mg. We size-matched the plerocercoids in all experimental triplets to avoid parasite size as a factor in mate choice, as *S. solidus* has been shown to prefer bigger mates [44]. The parasites were then placed in fork-shaped nylon mesh bags in a randomized order. This experimental setup was previously used and is described in further detail in Lüscher and Wedekind [44]. Briefly, the mesh bags consisted of three compartments separated by seams (Fig. 1). The focal worm was placed in the middle prong while the stimulus worms were placed in the two side prongs. All three openings of the bags were closed by carefully melting the nylon ends with a flame. Each bag was then placed in a glass container filled with approx. 400 ml of culture medium [40, 41] pre-warmed to 40 °C and covered with a lid to avoid evaporation. At the start of the experiment, the containers were placed in an incubator equipped with weak red light and a camera set and recording was started 15 min after the container was placed in the experimental chamber.

Mate choice trials ran for 48 h, and a picture was taken once every minute. This time frame includes maturation and egg production for *Schistocephalus* in vivo [38] as well as in vitro [40]. All cestodes were only used once in the experiment. In summary we recorded and evaluated 14 mate choice trials (seven with *S. pungitii* and seven with *S. solidus* as the focal worm) and five control trials with just two worms (one as a stimulus worm and the other as a focal worm, always of the same species).

While the stimulus worms could not leave their compartment, the focal worm could position itself anywhere. As the decisive criteria, the largest part of the focal worm’s body (>50 %) was assigned to one of the five positions at each minute (−2, −1, 0, 1 or 2, Fig. 1), or, if more than 25 % of two worms overlapped, this was counted as a possible mating attempt and classified with “-3” or “3”. One blind observer regarding the experimental setup carried out the scoring of all positions. After all scores were assigned and all failed trials (in some cases one of the worms managed to escape from the mesh bags) were excluded, we assigned parasite identity. We then classified all positive scores (1, 2 or 3) to the side with the conspecific and all negative scores to the side with the parasite from the different species (−1, −2 and −3).

### Statistical Analysis

We analyzed the data from the mate choice trials by evaluating whether the proportion of time the focal worms spent in each compartment differed between the three possible positions (same species for positive scores, neutral for 0 and different species for negative scores). We first assessed for the control, whether the focal worms spent more time with a conspecific than in empty compartments, then whether the focal worms in the dichotomous mate choice trials spent more time with a conspecific than in the neutral compartment or with a parasite from a different species and finally, whether the focal worms spent more time in a possible mating position (3 or −3 respectively) with a conspecific or a parasite from the different species. We used a square root transformation to achieve a normal distribution in the response variable (proportion of time spent in each compartment).

We first analyzed the data using a full-factorial ANOVA with the position and focal worm species as factors. As the focal worm species was not a significant factor (*p* > 0.05) in any of the models, it was excluded from the further analysis. Post-hoc Tukey HSD tests were used to compare the three different positions (same species, neutral or different species) to each other. The etasq function (heplots package, [45]) was used to estimate the effect size of our ANOVAs. Since we have used two independent tests on the same dataset (time spent on each side as well as time spent in a possible mating position) in the dichotomous mate choice trials, the threshold for significance was changed to *p* < 0.025 in these analyses to correct for multiple testing. All statistics were carried out using R version 3.2.4. [46].

## Results

### Natural hybridization in allopatric and sympatric species pairs

**Fig. 2:**
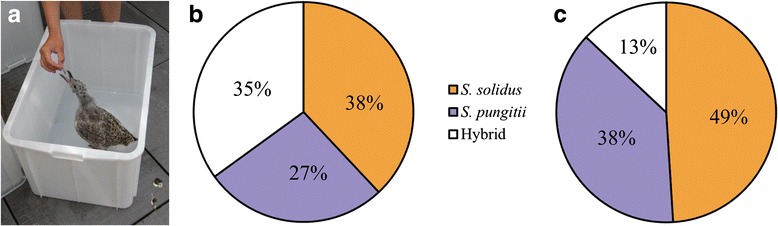
Hybridization frequencies of *S. solidus* and *S. pungitii* in their natural host. **a** Infection of lab-reared herring gulls and distribution of species in (**b**) allopatric and (**c**) sympatric combinations, determined by microsatellite typing of tapeworm larvae (*n* = 82 for each of 2 gulls) hatched from eggs isolated from the gull droppings

We collected eggs from the feces of both exposed herring gulls and hatched coracidia for an estimate of hybridization using microsatellite markers. In both cases the DNA from 82 out of 96 collected coracidia could be used for microsatellite typing, while in 14 cases per gull the amount or quality of the DNA was insufficient for usage. We detected hybrids from both herring gulls, with 35 % hybrid offspring in the gull exposed to the parasites of the allopatric, and 13 % hybrid offspring in the gull exposed to the parasites of the sympatric combination (Fig. 2). Even though the distribution of offspring (the proportion of *S. solidus*, *S. pungitii* or hybrids) differed between the two gulls, a quantitative conclusion cannot be made due to the fact that only one gull was exposed in each case.

We have no data on how many worms successfully established in each herring gull because dissection of the birds was not covered by our permission for this animal experiment. However, the experiment clearly showed that the hybridization between *S. solidus* and *S. pungitii* is not a laboratory artefact and that both worm species shared the same microhabitat within the gut of the herring gull for a time span long enough to enable a successful cross-species mating.

### Mate choice

**Fig. 3:**
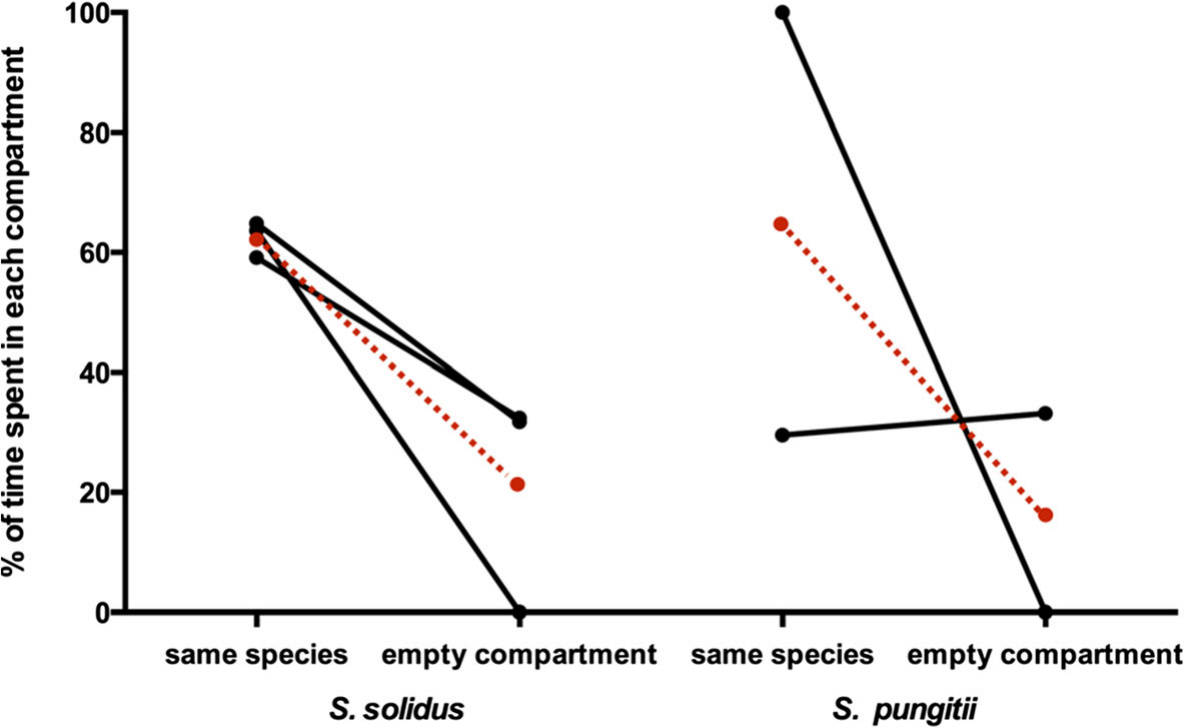
Mean percentage of time spent in the different compartments in control trials. For each focal worm species (*S. solidus*, *n* = 3 or *S. pungitii*, *n* = 2) the mean percentage of time on each side (same species: 1, 2 or 3, neutral: 0 (not shown), empty compartment: −1, −2 or −3, see Fig. 1) was calculated for each trial and the percentage of time the focal worm spent on either the side with the conspecific or in the empty compartment are shown. The red dots and dashed lines indicate the mean for each focal species. In both species, the focal worms spent more time on the side that contained a conspecific than in the neutral or empty compartment. The data do not add up to 100 % since time spent in the neutral compartment is not depicted for simplicity

Worms chose a conspecific over an empty compartment in the control experiment. We saw significant differences between the three assigned sides (worm, neutral, no worm) (ANOVA, F_2,12_ = 5.046, *p* < 0.05, η^2^ = 0.46, Fig. 3). Worms spent significantly more time on the side with a conspecific worm than in the neutral or empty compartment (post hoc Tukey HSD, *p* < 0.05).

**Fig. 4:**
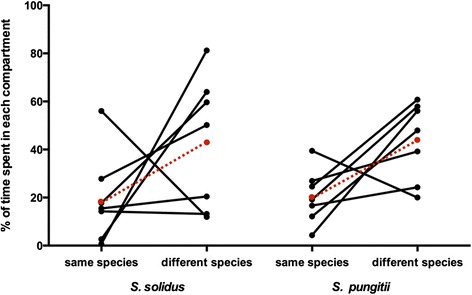
Mean percentage of time spent in the different compartments. For each focal worm species (*S. solidus*, *n* = 7 or *S. pungitii*, *n* = 7) the mean percentage of time on each side (same species: 1, 2 or 3, neutral: 0 (not shown), different species: −1, −2 or −3, see Fig. 1) was calculated for each trial and the percentage of time the focal worm spent on either the side with the conspecific or on the side with a different species are shown. The red dots and dashed lines indicate the mean for each focal species. The focal worms spent significantly more time on the side with a different species than with a conspecific. The data do not add up to 100 % since time spent in the neutral compartment is not depicted for simplicity

The focal worms in the dichotomous mate choice trials showed significant differences in the proportion of time spent in each compartment (ANOVA, F_2,39_ = 7.022, *p* < 0.025, η^2^ = 0.26, Fig. 4). A post hoc Tukey HSD test showed that the focal worms spent more time in the compartment with a different worm species than with the conspecific (*p* < 0.025), but also more time in the neutral compartment than with a conspecific (*p* < 0.025).

In summary, the majority of both *S. solidus* and *S. pungitii* focal worms spent on average more time in the compartment that contained a worm from a different species than in the compartment that contained a conspecific.

**Fig. 5:**
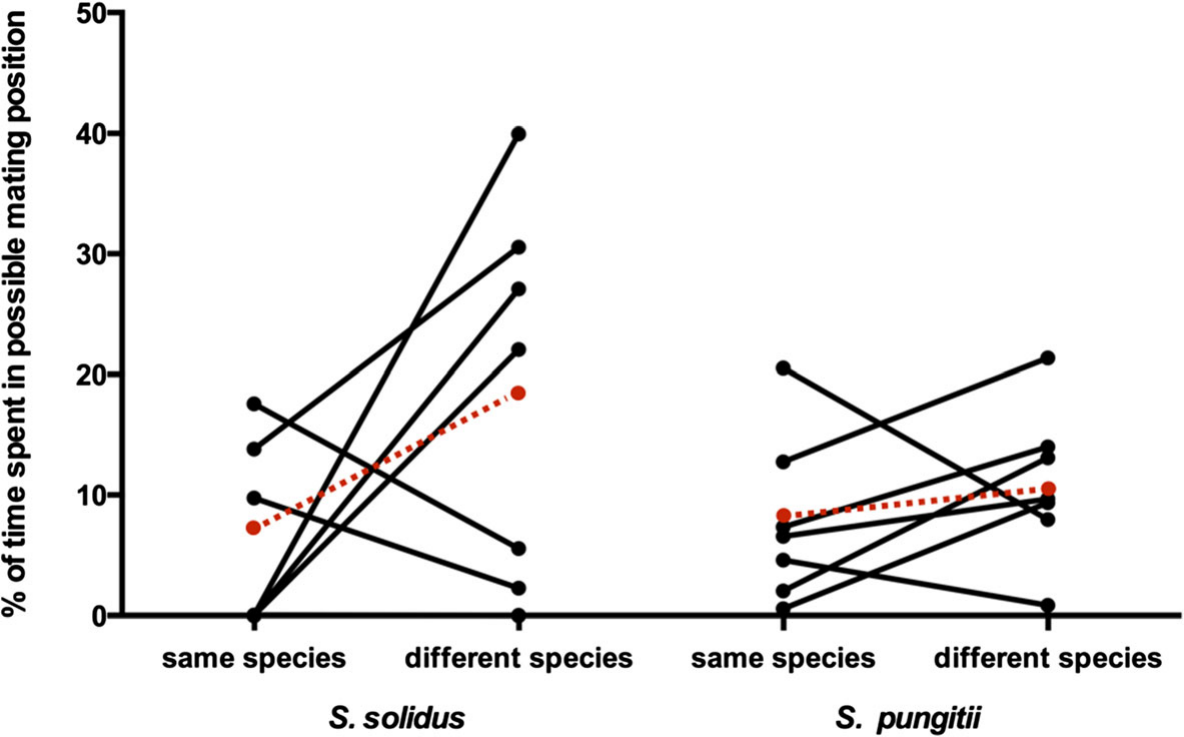
Mean percentage of time spent in a possible mating position. For each focal species (*S. solidus*, *n* = 7 or *S. pungitii*, *n* = 7) the mean percentage of time in a possible mating position (conspecific: 3, different species: −3, see Fig. 1) was calculated for each trial and the percentage of time the focal worm spent in a possible mating position with either the conspecific or the worm from a different species are shown. The red dots and dashed lines indicate the mean for each focal species. The focal worms spent significantly more time in a possible mating position with a worm from a different species than with a conspecific. The data do not add up to 100 % since time spent in positions other than the possible mating positions are not depicted for simplicity

We also used our experimental setup to evaluate the percentage of time each focal worm spent in a possible mating position with one of the stimulus worms, as this is a strong indicator for actual mate choice. Our results suggest that there is a strong trend for the focal worm to spent more time in a possible mating position (−3 or 3 respectively) with a worm from a different species, than with a conspecific (ANOVA, F_1,26_ = 4.089, *p* = 0.0536, η^2^ = 0.14, Fig. 5).

*S. pungitii* spent 7.79 % (±2.60 %) of the total time in a possible mating position with a conspecific and 10.92 % (±2.37 %) with the worm from a different species while *S. solidus* spent 5.88 % (±2.90 %) of the total time in a possible mating position with a conspecific and 18.21 % (±5.90 %) with the worm from a different species. Except for two worms in the control trial (where there was only one stimulus worm), all focal worms visited both sides during the mate choice trial. All *S. pungitii* focal worms spent time in a possible mating position with both stimulus worms while only 3 out of 7 *S. solidus* focal worms spent time in a possible mating position with both stimulus individuals. Three of the other 4 *S. solidus* focal worms only spent time in a possible mating position with a stimulus worm of the different species while the other spent only time in a possible mating position with a conspecific.

The detailed profiles of each focal worm’s location over time can be found in the Additional file 1: Figure S1.

## Discussion

The results from our experiments indicate that neither temporal (different timing of reproduction in the definitive host) nor spatial constraints (different location in the bird’s gut) hamper the two *Schistocephalus* species from mating in a natural definitive host. Hybridization in natural populations is therefore possible, if birds feed on sticklebacks infected with both cestode species. We assume that birds feeding on infected sticklebacks are unlikely to discriminate between *G. aculeatus* and *P. pungitius*. Even though both stickleback species inhabit slightly different ecological niches (with *P. pungitius* preferring more vegetated areas and *G. aculeatus* roaming more in the open water [47, 48])﻿, there is still an overlap in both species’ habitat and they are often caught together within the same shoal [49, 50]. For example, in one population used in this study from Obbola (Sweden), both stickleback species were caught with the same method (seine fishing) in the same net. We therefore infer that it is very likely that piscivorous birds prey on both stickleback species at the same time. Since the prevalence of *S. solidus* and *S. pungitii* in this population is very high for both stickleback species (70-90 %, unpublished data), we consider the likelihood of the two cestode species ending up in the same definitive host to be relatively high. Furthermore, since both *Schistocephalus* species have a broad range of definitive hosts and were recorded from a huge variety of naturally infected piscivorous birds [38, 51] and even from seals [52], we can exclude differences in susceptibility of definitive bird hosts as a possible source of reproductive isolation.

Our results also indicate no obvious barriers to hybridization in sympatric populations of the two *Schistocephalus* species. Even though the hybridization rate in the herring gull infected with a sympatric combination of plerocercoids was slightly lower, we could still detect hybrid offspring, which still gives us a qualitative answer: strong prezygotic barriers that would prevent hybridization have not evolved in this population in which both species occur in sympatry. In some cases, prezygotic barriers can arise in sympatric populations that prevent the formation of viable hybrids (e.g., reviewed in [53]). The mechanism by which prezygotic barriers arise can be a by-product of divergence [3, 54, 55], or hybridization can occur frequently, but hybrids are relatively unfit compared to their parental lines, and therefore outcompeted, which can lead to reinforcement [7]. Whether the hybrids from sympatric combinations have a lower fitness than hybrids from allopatric combinations was not tested in this experiment.

In the mate choice experimental setup actual mating is impossible since two layers of mesh separate the worms. Nevertheless, the focal worms showed a clear preference to spend more time close to a stimulus worm than in empty control compartments. *Schistocephalus* do not grow anymore in the definitive host, but become sexually mature within 25–30 h [38], therefore, there is virtually no reason for approaching a second worm, other than mating. It is rather surprising that our results indicate a preference of both parasite species for a parasite of a different species over conspecifics. It has been shown that *S. solidus* prefers to mate with siblings over a more distantly related conspecific [43]. Our results point in a completely different direction, indicating that the parasites might prefer maximal genetic distance in their mating partners, not taking species boundaries into account. Our system allowed multiple mating attempts and most parasites visited both stimulus worms during our trial (Additional file 1: Figure S1). However, we conclude that the total percentage of the time spent on one side is a good indicator for mate choice. This has also been previously shown by Lüscher and Wedekind [44] who demonstrated that *S. solidus* discriminates between sizes in their mating partners, preferring bigger mates. The total time span of 48 h for recording the worms’ movements was sufficient to cover the period until the peak in egg production was reached, based on previous experience with breeding worms from both species under similar conditions in an in vitro system.

The T-maze shaped net bag floating in a chamber with sterile cell culture medium is presumably quite different from the chemical, microbial and physical condition in the gut of a piscivorous bird. To what extent the cestodes’ mating preferences in the experiment actually resemble their natural behavior remains speculative. In case of a negative result i.e., no preference for either worm/side it would have been impossible to exclude an inhibitory effect of compounds from the culture medium or an artifact due to a poor simulation of the natural environment. However, since the focal worms did show consistent preferences for a worm over the empty control side or over another worm species, it is conservative to assume that there is no discrimination against different species. Therefore, in combination with the evidence from the gull infection experiment, we can state that assortative mate choice is not a basis for reproductive isolation between both *Schistocephalus* species.

Both species, *S. solidus* and *S. pungitii*, are simultaneous hermaphrodites and capable of self-fertilization. This mechanism ensures the parasite’s reproduction in case they do not find a suitable mating partner in the definitive host. Selfing has been shown to be costly, with self-fertilized offspring showing lower hatching rates [56–58] as well as lower infection and growth rates than outcrossed offspring [56, 59]. Negative fitness costs of self-fertilization mainly occur early in the parasite’s lifetime [60], and in sum lead to a lower probability of self-fertilized offspring reaching the age of sexual maturity. We could show in earlier experiments, that despite a lower hatching rate in hybrid crosses (which may be caused by a high proportion of self-fertilized offspring), hybrid cestodes that did hatch did not perform worse in their intermediate hosts, in both terms of infection rate and growth in the stickleback, than the parental species [36]. As hybrids are able to infect both stickleback species, they even extend their host range and therefore increase the probability of transmission to a suitable second intermediate host. Arguably, the high costs of self-fertilization may be one of the reasons why we can observe hybridization between these two taxa, as hybrids may be fitter than self-fertilized offspring.

It is possible that hybridization occurs frequently in some populations but has not been detected yet. Studies on gene flow of different species or populations of *Schistocephalus* are still rare [35] and should also consider the possibility of gene flow between these two species in the future. According to our results, there is no evidence for an efficient prezygotic reproductive barrier. Therefore, the role of postzygotic isolation in the speciation of the *Schistocephalus* should be fairly strong. So far we do not know if *Schistocephalus* hybrids are sterile or if other genetic incompatibilities would lead to an F2 hybrid breakdown. This point warrants further investigation.

## Conclusions

In summary, we did not find any prezygotic mechanisms that could prevent hybridization between *S. solidus* and *S. pungitii*, which emphasizes the potential importance of postzygotic barriers to reproduction in speciation of parasites that reproduce in the same definitive host. Additionally, this study highlights the importance of the ecological selection factors when studying speciation and the evolution of the barriers to reproduction. Prezygotic barriers in other species pairs of parasites may often arise as a side effect, if divergence of species is driven by the adaptation to different hosts that are present at the same time and location of reproduction. In our case, the specificity to different intermediate hosts probably does not lead to different definitive hosts, so that the two species may still frequently encounter each other in the definitive host.

## Additional file

Additional file 1: Figure S1. Individual profiles of the position of each focal worm in the mate choice experiment. *S. pungitii* was the focal worm in trials a-g (purple), *S. solidus* in trials h-n (blue) and the controls are trials m-s (green). Positive scores for the position indicate the location of the conspecific, negative scores indicate the position of the worm from a different species or empty compartments (for controls only). (DOCX 526 kb)
