# Supplementary material for: The role of prezygotic isolation mechanisms in the divergence of two parasite species
Source: BMC Evol Biol. 2016 Nov 9;16:245. doi: 10.1186/s12862-016-0799-5 (PMC5103353; doi:10.1186/s12862-016-0799-5)
Supplement: Additional file 1: Figure S1. — Individual profiles of the position of each focal worm in the mate choice experiment. S. pungitii was the focal worm in trials a-g (purple), S. solidus in trials h-n (blue) and the controls are trials m-s (green). Positive scores for the position indicate the location of the conspecific, negative scores indicate the position of the worm from a different species or empty compartments (for controls only). (DOCX 526 kb) [file 12862_2016_799_MOESM1_ESM.docx]

**Additional file 1**

Figure S1

a)

b)

c)

d)

e)

f)

g)

h)

i)

j)

k)

l)

m)

n)

o)

p)

q)

r)

s)

**Figure S1: Individual profiles of the position of each focal worm in the mate choice experiment**

*S. pungitii* was the focal worm in trials a-g (purple), *S. solidus* in trials h-n (blue) and the controls are trials m-s (green). Positive scores for the position indicate the location of the conspecific, negative scores indicate the position of the worm from a different species or empty compartments (for controls only).
